# Self-Regulation, Emotional Symptomatology, Substance Use, and Social Network Addiction in Adolescent Self-Harm

**DOI:** 10.3390/bs15030257

**Published:** 2025-02-23

**Authors:** Luis Fernando López-Martínez, Eva M. Carretero, Miguel A. Carrasco, Ana M. Pérez-García

**Affiliations:** 1 Faculty of Psychology, Universidad Nacional de Educación a Distancia (UNED), 28040 Madrid, Spain; luisfernandolopez@isniss.es (L.F.L.-M.); evacarretero@isniss.es (E.M.C.); macarrasco@psi.uned.es (M.A.C.); 2 Ph.D. Program in Psychology, Escuela Internacional de Doctorado de la UNED (EIDUNED), 28040 Madrid, Spain; 3 Faculty of Psychology, Universidad Complutense de Madrid, 28223 Pozuelo de Alarcón, Madrid, Spain

**Keywords:** non-suicidal self-injury (NSSI), self-regulation, social network addiction, substance use, adolescents, gender

## Abstract

Background: Non-suicidal self-injurious behaviour (NSSI) is a growing concern in the field of adolescent mental health. It is thus crucial to examine the factors associated with this behaviour. Methods: A sample of 354 adolescents (51.7% boys), with a mean age of 15.01 years (ranging from 12 to 20 years), was analysed to explore the relationships between NSSI and self-regulation strategies, substance use, dependence on social networks, and symptomatology. Results: The results indicate that adolescents who engage in NSSI employ fewer adaptive emotional self-regulation strategies and exhibit more self-blame and rumination than those who do not engage in NSSI. Additionally, these adolescents show greater psychological symptomatology, more dependence on social networks, and increased substance use. Gender is also an important factor, with more girls (62.8%) than boys (37.2%) engaging in self-harm. Regression analyses show that self-harm is associated with greater symptomatology and increased substance use for boys and girls alike. For girls, self-harm is also associated with maladaptive self-regulation strategies. The variables chosen for analysis allowed us to correctly classify 89.5% of the boys who did not self-harm and 72.8% of the girls who did. Conclusions: This study offers insight into the relationships among self-regulation, digital addiction, substance use, emotional symptomatology, and NSSI in adolescents, highlighting the importance of gender.

## 1. Introduction

Non-suicidal self-injury (NSSI) is particularly relevant during adolescence, as this is a critical period for emotional and behavioural development. During adolescence, biopsychosocial changes not only amplify individual vulnerabilities but also create an environment conducive to the emergence of maladaptive behaviours such as NSSI (González-Arrimada et al., 2023). Several studies have reported an increase in the incidence of these behaviours among adolescents, with prevalence rates ranging from 4% to 45%, showing a higher frequency in girls than in boys and in clinical samples compared to general population samples (De Luca et al., 2023; Duarte et al., 2021; Faura-Garcia et al., 2021; González-Arrimada et al., 2023).

Similarly, studies on the Spanish population of adolescents and young adults have reported prevalence rates ranging from 10.3% up to half of the samples studied (Calvete et al., 2015; Carrasco et al., 2023). Alongside adolescence, gender emerges as another significant risk factor. Being female, especially in the 15–25 age range, is strongly associated with factors such as biographical stress and family conflicts, which in turn are linked to NSSI (Fleta Zaragozano, 2017; McEvoy et al., 2023; Schmidt et al., 2023; Young et al., 2014).

These findings underscore the importance of studying this phenomenon in the adolescent population, especially among girls. They highlight not only the seriousness of the issue, but also the need to identify the most relevant variables associated with NSSI among adolescent girls. This will be crucial for the development of prevention and early detection strategies tailored to the specific needs of this age group.

NSSI is a multifactorial phenomenon of remarkable complexity (Nock, 2010), associated with the confluence of various risk factors. These include emotional conditions such as depression and anxiety (Duarte et al., 2021; Flores-Soto et al., 2018; Ramírez Gamboa & Restrepo Soto, 2022), adolescents’ own self-regulation (Bautista Hernández et al., 2022; Ospina Gutiérrez et al., 2019; Sánchez-Sánchez, 2018), risk behaviours associated with substance use (Del Brío Ibáñez et al., 2019; Faura-Garcia et al., 2021; Obando et al., 2018), and contextual factors associated with problematic internet and social network use (Gámez-Guadix et al., 2020; López-Martínez, 2020).

Problematic social network use has been linked to increased anxiety, depression, and maladaptive emotional regulation strategies, all of which are closely associated with NSSI (Gámez-Guadix et al., 2020; Troya-Fernández et al., 2023). Similarly, exposure to self-harm-related content and the search for online validation may reinforce the persistence of these behaviours, particularly in emotionally vulnerable adolescents (Carretero et al., 2024). Additionally, substance use has been identified as a relevant factor: it can exacerbate emotional distress and impulsivity, further intensifying self-injurious behaviours (Jeréz-Cañabate et al., 2023; Ramírez Gamboa & Restrepo Soto, 2022).

While the significant association of these variables with NSSI has already been established, further research is needed to jointly analyse these variables on self-injurious behaviours and explore their differential and relative contributions to the phenomenon, as well as the interplay between them. For example, maladaptive regulation strategies such as self-blame or rumination tend to perpetuate emotional distress in adolescents (Casas Dorado, 2016; Flores-Soto et al., 2018; Sánchez Sánchez, 2017), facilitating the presence of NSSI as a means of alleviating or avoiding negative emotions, expressing suffering or distress, or seeking a sense of control over inner emptiness. A lack of adequate tools to manage emotions contributes significantly to the onset and maintenance of NSSI (Aizcorbe & Gallo, 2024; Fleta Zaragozano, 2017; Greenberg et al., 2022; Klonsky, 2007; Mollà et al., 2015; Sánchez Alonso, 2021).

Emotional distress has also been shown to facilitate substance use (Ferreiro et al., 2023; Jeréz-Cañabate et al., 2023) and problematic use of the internet or social networks (Freitas et al., 2021; Guzmán Brand & Gélvez García, 2023; Regalado Chamorro et al., 2022), which in turn are associated with NSSI (Carretero et al., 2024; López-Martínez, 2020). Similarly, excessive internet and social network consumption has been linked to both emotional distress in adolescents (Arab & Díaz, 2015; Regalado Chamorro et al., 2022; Troya-Fernández et al., 2023) and the practice of NSSI (Gámez-Guadix et al., 2020; Garitaonandia et al., 2020). This evidence underscores the importance of jointly analysing these variables.

In line with the background discussed above, the general aim of the current study was to analyse the relationships between NSSI and the variables of self-regulation, emotional problems, substance use, and social network addiction. More specifically, the following objectives were established: (1) to analyse adolescents with and without NSSI in terms of these variables, examining the associations between each variable and NSSI; (2) to explore how these factors may explain the severity of NSSI and develop a model to account for its occurrence; (3) to determine whether this set of factors can predict and classify adolescents with NSSI versus those without NSSI, while assessing the specific predictive contribution of each factor; and (4) to examine the role of sex in the relationships between these variables and NSSI.

Based on the reviewed literature and the study’s objectives, we expect to find significant associations between NSSI and a set of key variables: emotional symptomatology, emotional self-regulation strategies, social network addiction, and substance use. Specifically, we anticipate that maladaptive self-regulatory strategies, along with high levels of anxiety, depression, and withdrawal, will be the most prominent predictors of NSSI. As numerous studies have shown, self-harm is associated with emotional distress and inadequate coping, with maladaptive regulatory strategies acting as an inadequate coping mechanism, which in turn contributes to increased distress and the perpetuation of NSSI in a vicious cycle. Further, gender is expected to appear as a significant factor in these relationships. Girls will likely show a significantly higher prevalence of NSSI compared to boys, as well as greater vulnerability to maladaptive self-regulation strategies and dependence on social networks (Arab & Díaz, 2015; Carretero et al., 2024; Faura-Garcia et al., 2021; Gámez-Guadix et al., 2020; González-Arrimada et al., 2023; Guzmán Brand & Gélvez García, 2023). Finally, we expect that the variables chosen for analysis will allow a significant differentiation to be made between adolescents with and without NSSI. The relative weight of each factor will allow more robust predictive models to be established, which will contribute to the adaptation of prevention and intervention strategies to individual and contextual characteristics.

## 2. Method

### 2.1. Participants

The convenience sample (non-random sample) was composed of 354 participants, ranging from 12 to 20 years old (*M* = 15.01 years, *SD* = 1.91). Of the participants, 51.7% were male (*n* = 183) and 48.3% were female (*n* = 171). In terms of place of origin, 83.9% were from Spain (*n* = 297) and 14.4% from outside Spain (*n* = 51). Data were missing for six cases in this category.

Regarding parental origins, 74.6% of fathers (*n* = 264) and 77.4% of mothers (*n* = 274) were of Spanish nationality. Various nationalities were observed among parents born outside Spain, including Venezuela (2.5%), Ecuador (3.1%), and Morocco (2.0%) among the fathers, and Ecuador (3.1%), Venezuela (3.1%), and Morocco (2.0%) among the mothers.

Within the sample, 70.3% of participants lived with both parents, 11.3% lived with one parent, 7.6% lived in shared custody, and 6.8% lived in households with one parent and their parent’s current partner.

We found that 94.6% of the young people in the sample reported having their own mobile phone, and of this group, 92.1% had an internet connection. Regarding time spent on social media and browsing the internet, 26.6% of the sample spent two hours online daily, while 41.5% exceeded three hours of daily online activity.

During the sample selection process, the following inclusion criteria were applied: Participants had to be enrolled in the analysed institutions, provide informed consent signed either by the participants themselves or by their legal guardians, and complete the administered assessments correctly. The exclusion criteria consisted of failing to meet any of the aforementioned requirements.

### 2.2. Instruments and Measures

The following section outlines the instruments used to measure the variables assessed in the current study; namely, self-injury, drug and social media addiction, self-regulation, symptomatology, and sociodemographic variables. The selected tests were chosen based on their widespread application in this field of study and their well-established psychometric properties, ensuring the reliability and validity of the measurements.

*Self-injury measures*. The *Self-Report Scale Functional Assessment of Self-Mutilation* (FASM) (Calvete et al., 2015; Lloyd et al., 1997) was used to evaluate the occurrence and frequency of different types of self-injurious behaviours. These behaviours include cutting or scratching the skin, hitting oneself, pulling out hair, picking at wounds, inserting objects under the skin, biting oneself, and rubbing or scratching the skin to the point of bleeding. In this study, tattooing was excluded as a form of self-injury, and the item related to tattoos was omitted when calculating the total self-injury score. The scale captures the methods, frequency, and functions of self-injury over the 12 months prior to the assessment. In the current sample, the scale demonstrated acceptable internal consistency by Cronbach’s alpha (11 items, α = 0.75). For analytical purposes, self-injury was considered both as a categorical variable (the presence of at least one self-injurious behaviour vs. the absence of self-injury) and as a continuous variable based on the total score indicating the intensity of NSSI.

*Social media and drug addiction.* These factors were assessed using two measures: (1) the Symptoms of Addiction scale, which evaluates addiction to online technologies (e.g., “I would be angry if I had to do without social networks”). This scale consists of 9 items (α = 0.73) and is derived from the *Scale of Risk of Addiction to Social Networks and Internet for Adolescents* (Peris et al., 2018). The ERAR-SI comprises 29 items distributed across four dimensions: symptoms of addiction, social media use, “freaky” traits, and nomophobia. Responses are recorded on a four-point frequency scale ranging from 1 (never or almost never) to 4 (almost always or always). (2) A single-item question was specifically developed for this study: “Have you ever taken any drugs other than alcohol?” Responses were measured on a 4-point Likert scale: 1 (“never”), 2 (“in isolation”), 3 (“from time to time”), and 4 (“often”).

*Self-regulation measures*. Three measures were used to assess self-regulation:

(1) The total score of adaptive regulation (13 items, α = 0.68) from *the Brief Self-Control Scale* (Del Valle et al., 2019; Tangney et al., 2004). This consists of a 13-item broad measure for assessing self-control, which includes three dimensions: non-reflective control of impulses, self-discipline, and reflective control of impulses. A 5-point Likert scale ranging from 1 (“not at all”) to 5 (“very much”) indicates how much a person typically agrees with each item (e.g., “I am good at resisting temptation”).

(2) The *self-blame scale* (four items, α = 0.67), which measures how much the respondent thinks that they are responsible for what happened.

(3) The *rumination scale* (four items, α = 0.76), which measures the respondent’s reflections, feelings and thoughts associated with what happened.

These scales are part of the *Cognitive Emotion Regulation Questionnaire (CERQ-S)* (Domínguez-Sánchez et al., 2013; Garnefski et al., 2001). This 36-item questionnaire measures cognitive emotional regulation strategies used in response to a stressful life event (i.e., Self-blame, Acceptance, Rumination, Positive refocusing, Refocusing on planning, Positive reappraisal, Putting into perspective, Catastrophising, and Other-blame). Items are measured using a Likert-type scale (from 1 = almost never, to 5 = almost always), with higher scores greater indicating the use of the coping strategy in question. Self-blame and rumination are considered less adaptive strategies.

*Psychopathological Symptoms*. The internalising broad dimension of the *Youth Self Report* (YSR) (Achenbach & Rescorla, 2001) was used (21 items, α = 0.87), which includes anxiety, depression, and withdrawal symptoms. YSR is a self-report questionnaire that evaluates emotional and behavioural problems in children and adolescents. It consists of 112 items measured on 3 Likert-type scale response options (0 = not true to 3 = true, very often, or fairly often).

*Socio-demographic data and additional information.* A sheet of sociodemographic and complementary information was developed ad hoc for this study, which included information about sex (boy versus girl), age, place of birth, and with whom the participant usually lived. Additionally, participants provided information about their possession of a mobile phone, internet access, social network usage, and time spent on these platforms.

### 2.3. Procedure

Data collection was conducted by two trained PhD researchers using a questionnaire that participants completed individually in their regular classrooms. The questionnaire was administered in a single session lasting approximately one hour, ensuring that all participants received consistent instructions on how to proceed.

The boards of the participating secondary schools and the university’s ethics committee approved the study, which was conducted in accordance with the ethical principles outlined in the Declaration of Helsinki.

To ensure compliance with ethical standards, informed consent was obtained from parents or legal guardians for participants under 16 years of age, and from the students themselves for those over 16. The participants were informed that their responses would be treated anonymously and confidentially, and that the results would be used exclusively for research purposes. The questionnaires were administered by the researchers, with support from the teachers present in the classrooms throughout the process.

### 2.4. Data Analysis

The study was quantitative and cross-sectional. Correlation and multivariate analysis of variance (MANOVA) analyses were conducted to examine the effects of sex (boys and girls) by NSSI (yes and no) on addiction (social networks and substance abuse), self-regulation (self-control, self-blame, and rumination), and psychological symptomatology. These analyses aimed to inform an understanding of the differences between boys and girls, as well as between adolescents with and without non-suicidal self-injurious behaviours, with respect to these variables.

To provide a more comprehensive understanding of the relationships between variables, two types of regression analyses were conducted: multiple regression, using NSSI as a continuous variable, and logistic regression, using NSSI as a categorical variable. Multiple regression was employed to explore how predictors or factors explain the severity of NSSI, as well as to assess the specific contribution of each predictor to the model. Logistic regression, on the other hand, was used to examine whether the set of factors could predict and influence the presence or absence of NSSI. The variables used as predictors were sex, addictions (social networks and substance use), adaptive (self-control) and maladaptive (self-blame + rumination) strategies of self-regulation, and psychological symptomatology. Finally, logistic regression analyses were performed to assess the impact of sex, addictions, strategies of self-regulation, and psychological symptomatology on the likelihood that adolescents would engage in self-injury.

## 3. Results

Preliminary sample data indicate that the prevalence of NSSI was higher among girls (62.8%) than boys (37.2%) [*X*^2^ (1, *N* = 354) = 21.51, *p* < 0.001; Cramer’s *V* = 0.247]. More than half of those who self-harmed (52.7%) logged into social networking sites for three or more hours per day. This percentage dropped to one-third (33.5%) among those who did not self-injure [*X*^2^ (2, *N* = 354) = 14.51, *p* = 0.001; Cramer’s *V* = 0.202]. On the other hand, among those who self-injured, 61.5% had high social network dependence (score above the median), compared to 64.6% of those with low dependence (score below the median) who did not self-injure [*X*^2^ (1, *N* = 354) = 23.50, *p* < 0.001; Cramer’s *V* = 0.258].

Table 1 shows the Pearson correlations between the analysed variables. All correlations are statistically significant, highlighting the variables’ relationships with NSSI (from *r* = 0.24 to *r* = 0.30) and psychological symptomatology (from *r* = 0.16 to *r* = 0.43). The association between NSSI and symptomatology accounted for 29% of the variance (*p* < 0.001). Also noteworthy is the correlation between the two maladaptive self-regulatory strategies of self-blame and rumination (*r* = 0.63; *p* < 0.001).

The means and standard deviations by sex (boys and girls) and NSSI for addiction to social networks and the internet, substance use, self-regulation, and psychological symptomatology are presented in Table 2. First, two MANOVAs were conducted: one to analyse the effects of NSSI and sex on addictive behaviours, and another to analyse the effects of NSSI and sex on self-regulation strategies.

The first MANOVA analysis revealed significant effects of NSSI (Wilks’ *Λ* = 0.94, *F* = 11.63, *p* < 0.001, *pη*^2^ = 0.064) and sex (Wilks’ *Λ* = 0.97, *F* = 5.93, *p* = 0.003, *pη*^2^ = 0.034) on addictions. Tests of the between-subjects effect showed a significant effect of NSSI on addiction to social networks (*p* < 0.001) and substance use (*p* = 0.011); and a significant effect of sex on addiction to social networks (*p* = 0.001). Girls scored significantly higher for addiction to social networks, while boys and girls exhibited similar scores for substance use. Adolescents who self-injured scored higher for both addiction-related behaviours: social networking and substance use. The effect size (Cohen’s *d*) for both independent variables (sex and NSSI -yes/no-) was small for substance use and medium for addiction to social networks (see Table 2).

The second MANOVA analysis identified significant effects of NSSI (Wilks’ *Λ* = 0.92, *F* = 10.33, *p* < 0.001, *pη*^2^ = 0.082) and sex (Wilks’ *Λ* = 0.97, *F* = 4.22, *p* = 0.006, *pη*^2^ = 0.035) on self-regulation strategies. Tests of the between-subjects effect showed a significant effect of NSSI on all three strategies under consideration: self-control (*p* < 0.001), self-blame (*p* = 0.002), and rumination (*p* = 0.018); and a significant effect of sex (*p* = 0.001) on rumination. Girls scored significantly higher for rumination, while boys and girls scored similarly for self-control and self-blame. Adolescents who self-injured scored lower on self-control and higher on rumination and self-blame self-regulatory strategies compared to those who did not self-injure. The effect size (Cohen’s *d*) when comparing boys and girls was small for self-control and self-blame, and medium for rumination. The effect size (Cohen’s *d*) when comparing NSSI (yes/no) was small for self-blame and rumination, and medium for self-control.

No significant interactions between sex and NSSI were found in any of the previous analyses (all with *p ≥* 0.05).

On the other hand, an ANOVA analysing the relationships between NSSI and sex on psychological symptomatology revealed that adolescents who self-injured reported more symptoms than those who did not self-injure (*p* < 0.001), and girls reported more symptoms than boys (*p* = 0.001). The effect size (Cohen’s *d*) of the differences in symptomatology was medium for the comparison between boys and girls, and large for the NSSI (yes/no) comparison. No significant interaction between sex and NSSI was found (*p ≥* 0.05).

Finally, the total score for the number of self-injuries was significantly higher for girls (*mean* = 1.74, *SD* = 2.09) than for boys (*mean* = 0.81, *SD* = 1.40), with a medium effect size [*F* (1, 353) = 24.06, *p* < 0.001; *d* = 0.52].

The modelling and prediction of non-suicidal self-injurious behaviour (NSSI) was analysed on the basis of addictions (social networks and substance use), self-regulation and psychological symptomatology. Given the high correlation between self-blame and rumination strategies (*r* = 0.63), and multicollinearity effects observed in previous analyses with both strategies considered as separated predictors, a single variable (the sum of both subscales) was developed to capture the joint score of maladaptive self-regulation strategies (*mean* = 21.74, *SD* = 6.89, *α* = 0.82, eight items).

The variables used as predictors were sex, addictions (addiction to social networks and substance use), adaptive (self-control) and maladaptive (self-blame + rumination) strategies of self-regulation, and psychological symptomatology (a score that includes anxiety, depression, and withdrawal).

In the multiple regression analysis, the regression model for the total sample was found to be statistically significant [*F* (6, 345) = 29.15, *p* < 0.001]. The multiple correlation coefficient (R), using all the predictors simultaneously, was 0.58 (R^2^ = 0.34), and the adjusted R^2^ was 0.33, indicating that 33% of the variance in self-injury could be predicted from the independent variables considered. Significant predictors included sex (with a higher prevalence of NSSI in girls), symptomatology, and substance abuse. The regression models were also significant for boys [*F* (5, 176) = 12.19, *p* < 0.001] and girls [*F* (5, 168) = 19.43, *p* < 0.001] when analysed separately, explaining 24% and 35%, respectively, of the NSSI. The maladaptive self-regulation strategies analysed (self-blame + rumination) were also significant predictors of NSSI for girls (see Table 3).

Finally, we performed logistic regression analyses to assess the impact of sex, addictions (social networks and substance use), strategies of self-regulation (self-control as an adaptive strategy and self-blame and rumination as maladaptive strategies), and psychological symptomatology on the likelihood that adolescents would engage in NSSI behaviours. Additionally, we examined the potential capacity of these predictors to classify adolescents into NSSI groups. The logistic regression model was statistically significant for the total sample [*χ*2 (6, *N* = 346) = 87.65, *p* = 0.0001], for boys [*χ*2 (5, *N* = 177) = 29.59, *p* = 0.0001], and for girls [*χ*2 (5, *N* = 169) = 46.40, *p* = 0.0001]. The models explained (Nagelkerke *R*^2^) 30%, 22%, and 32% of the variance in NSSI for the total sample, boys, and girls, respectively. The models correctly classified 69.7% (79.6% no-self-injury, 55.9% self-injury) of cases for the total sample; 72.3% (89.5% no-self-injury, 32.1% self-injury) of cases for boys; and 68% (62.3% no-self-injury, 72.8% self-injury) of cases for girls. Table 4 presents the coefficients and logistic regression statistics.

Two factors were found to be significant predictors of the presence/absence of self-injury in the total analysed sample: sex and psychological symptomatology. The use of maladaptive self-regulation strategies (self-blame and rumination) was also a significant predictor of self-injury among girls. Addictions (to social network and substance use) and the use of adaptive self-regulation strategies (self-control) were not significantly associated with the presence/absence of self-injury among the adolescents in the sample.

The odds ratios (ORs) indicate that the probability of engaging in self-injury increased 1.12 times for each point increase in psychological symptomatology. Additionally, being a girl increased the probability of self-injury by 1.90 times, and using maladaptive self-regulation strategies (self-blame and rumination) increased the probability of self-injury by 1.07 times in girls.

## 4. Discussion

The high prevalence of NSSI among adolescents (Carrasco et al., 2023; Carretero et al., 2024; Ferreiro et al., 2023; Mollà et al., 2015; Ospina Gutiérrez et al., 2019; Sánchez-Sánchez, 2018; Villarroel et al., 2013) has emerged as an increasing concern in the field of mental health. Gaining a deeper understanding of the factors associated with this phenomenon remains a critical challenge in contemporary research, highlighting the need for further studies and robust empirical evidence. The primary aim of this study was to examine the relationships between NSSI and a set of variables, including addictions (e.g., social network use and substance use), self-regulation (adaptive strategies such as self-control, and maladaptive strategies such as self-blame and rumination), and psychological symptomatology. As expected, the findings reveal significant associations between NSSI and maladaptive self-regulation strategies (self-blame and rumination), elevated levels of anxiety, depression, and withdrawal symptoms, as well as greater dependence on social networks and psychoactive substances. Moreover, these relationships exhibited notable differences between boys and girls that should be considered.

A significant difference in the prevalence of NSSI with regard to gender was observed, with a higher proportion of cases among girls (62.8%) compared to boys (37.2%). This finding is consistent with the existing literature, which indicates that self-harm is more common among females than males (Carretero et al., 2024; Faura-Garcia et al., 2021; González-Arrimada et al., 2023).

The first objective of the study was to explore differences between adolescents with and without NSSI. In terms of addictive behaviours, boys and girls who self-harmed exhibited higher levels of addiction to social networks and substance use. This finding aligns with studies by Ospina Gutiérrez et al. (2019) and Troya-Fernández et al. (2023), who identify these behaviours as maladaptive strategies of emotional regulation. Similarly, Jeréz-Cañabate et al. (2023) highlight that both NSSI and substance use serve as emotional relief mechanisms, particularly among adolescents experiencing high levels of distress. However, Sánchez-Sánchez (2018) notes that these associations may vary across cultural and social contexts, suggesting that they are not universally observed. Overall, the evidence supports findings in the literature that emphasise the strong connection between emotional dysregulation, NSSI, and addictive behaviours.

Differences in self-regulation strategies were also observed, with boys and girls who engaged in NSSI showing less self-control and utilising more self-regulatory strategies, such as rumination and self-blame, compared to adolescents who did not self-harm. This finding aligns with the work of Bautista Hernández et al. (2022), who highlight the connection between emotional dysregulation, low self-control, and maladaptive coping mechanisms in adolescents with NSSI. Similarly, Sánchez-Sánchez (2018) emphasise that self-blame and rumination are common among adolescents with emotional distress. However, some studies, such as Jeréz-Cañabate et al. (2023), indicate that not all adolescents with NSSI show significant differences in self-regulation when psychiatric co-morbidities are controlled for, indicating variability across populations. Overall, this evidence supports the link between poor self-regulation and NSSI, consistent with the emphasis in the literature on emotional vulnerability and maladaptive coping strategies.

Adolescents who engaged in self-harm also exhibited more acute psychological symptoms compared to those who did not engage in self-injurious behaviour. This finding is consistent with previous research, such as Carrasco et al. (2023), which underscores the relationship between self-injurious behaviours, both suicidal and non-suicidal, and various mental health issues, including depression and anxiety. Similarly, Carretero et al. (2024) highlight how emotional vulnerability and impulsivity contribute to the higher prevalence of symptoms among adolescents who self-harm. Other studies, such Sánchez Alonso (2021), point out that the variability of symptomatology depends on diagnostic criteria, contextual factors, and the presence of comorbidities. Overall, this evidence is consistent with the existing literature that links NSSIs to increased psychological distress, reinforcing the need for early intervention and comprehensive assessments.

The effect sizes of these differences ranged from small to medium, except for the large effect size differences observed in symptomatology between adolescents with and without NSSI.

To explore how these factors may explain the severity of NSSI (objective two), three significant variables were identified: sex (with a higher presence of NSSI in girls), symptomatology, and substance abuse. Together, these factors accounted for up to 33% of the variance in self-injury. Of these, symptomatology (i.e., depression, anxiety, and withdrawal) emerged as the most important predictor, surpassing sex and substance abuse.

Building on these findings, the studied factors were also found to be capable of significantly predicting and classifying adolescents with or without NSSI (objective three). In line with the multiple regression analyses, logistic regression revealed that two factors were significant predictors of the presence or absence of self-harm in the total analysed sample: gender and psychological symptomatology. However, substance abuse did not significantly differentiate adolescents with and without NSSI. The ORs indicate that the probability of self-harm increased by 1.12 times for each point increase in psychological symptomatology. These variables correctly classified 69.7% of cases in the total sample (79.6% no-self-injury, 55.9% self-injury).

These findings reaffirm psychological symptomatology—specifically depression, anxiety, and withdrawal—as the most significant predictor of NSSI severity, consistent with previous studies (Hamza et al., 2015; Nock et al., 2006). Symptomatology explained a significant proportion of the variance in self-harm, underscoring its central role in understanding these behaviours. In contrast, while substance abuse contributed to the severity of NSSI in the multiple regression analysis, it did not differentiate adolescents with and without NSSI in the logistic regression analysis. This suggests that, as noted by Obando et al. (2018), while substance use may intensify self-injurious behaviours, it lacks the specificity to serve as a primary predictor, especially compared to other variables like impulsivity (Carretero et al., 2024).

The results also indicate that maladaptive self-regulation strategies, such as self-blame and rumination, are more significant predictors of NSSI than adaptive strategies. These patterns, which are especially prevalent in girls, not only increase emotional distress but can also perpetuate and reinforce NSSI, as suggested by Sánchez-Sánchez (2018). In the present study, logistic regression emphasised the importance of symptomatology and gender in classifying adolescents, with the models correctly identifying nearly 70% of cases. Notably, maladaptive strategies were predictive only for females, aligning with studies that report higher emotional vulnerability and a greater prevalence of NSSI among girls (Carretero et al., 2024).

The finding that substance use is not a significant differentiating factor between adolescents with and without self-harm, despite its contribution to explaining the severity of NSSI in multiple regression models, may be linked to the widespread prevalence of substance use in this population. Research by Obando et al. (2018) and Ramírez Gamboa and Restrepo Soto (2022) suggests that substance use is common among adolescents, regardless of whether they engage in self-harm. This general habit may reduce the substance use variable’s ability to distinguish between adolescents who self-harm and those who do not.

In the context of multiple regression, where NSSI was measured continuously as an indicator of severity, substance use appeared as a significant factor, likely due to its role in amplifying emotional and behavioural difficulties (Carrasco et al., 2023; Ferreiro et al., 2023). However, in dichotomous classification models (with/without NSSI), its impact was diminished, suggesting that its relevance lies more in the aggravation of self-injurious behaviours rather than in their initial identification. This finding is consistent with previous studies highlighting the non-specific role of substance use as a risk factor associated with multiple psychological and behavioural issues in adolescents (Carretero et al., 2024; Del Brío Ibáñez et al., 2019).

Additionally, the impact of substance use may be related to the concurrent use of maladaptive coping strategies, such as rumination and self-blame, which heighten emotional distress and self-harm (Bautista Hernández et al., 2022; Sánchez-Sánchez, 2018). These interactions may help to explain why substance use does not effectively discriminate between adolescents with and without self-harm but rather intensifies the severity of these behaviours.

From a practical perspective, these results suggest that substance use, although relevant to understanding the severity of NSSI, is not an adequate variable to discriminate between adolescents with and without these behaviours. This has important implications for clinical assessment. On the one hand, it emphasises the need to combine more specific factors, such as emotional symptomatology and self-regulation strategies, to improve the identification of at-risk adolescents. On the other hand, it highlights the role of substance use as a secondary factor exacerbating NSSI rather than acting as an initial marker.

This reinforces the importance of a comprehensive approach to assessment and intervention in which variables such as emotional symptomatology and maladaptive coping strategies play a central role. Moreover, it suggests that prevention strategies should focus not only on reducing substance use, but also on promoting emotional skills that mitigate its amplifying impact on self-harm.

An important aim of this study was to examine the role of gender in the relationships between self-harm and addictions, self-regulation, and emotional symptoms (objective four). The findings reveal that sex is a significant variable in terms of differences in how predictors explain NSSI and impact the classification of adolescents with or without NSSI. In particular, girls demonstrated significantly more addiction to social networks than boys, were more likely to use rumination as a maladaptive self-regulation strategy, and reported more internalising symptoms. Furthermore, no significant interactions were observed between sex and NSSI, suggesting that sex is more than just a moderator of the relationships between NSSI and other factors; sex itself can explain these behaviours. For example, less adaptive self-regulation strategies, such as self-blame and rumination, explained NSSI only in girls, not boys, and the use of maladaptive self-regulation strategies (self-blame and rumination) increased the probability of self-injury by 1.07 times in girls (not boys). As a result, the developed model explained a higher percentage of the variance in NSSI and also had greater capacity to classify girls according to the presence of NSSI than it did for boys.

The findings are consistent with the existing literature, highlighting the relationship between self-injurious behaviour (NSSI) and mental health problems such as depression and anxiety. Moreover, it underscores the importance of associated psychological factors, such as dysfunctional self-regulatory strategies like rumination and self-blame, which have been identified in the literature as elements linked to internalising symptoms that contribute to the development of these behaviours (Carrasco et al., 2023; Sánchez-Sánchez, 2018). These behaviours reflect a greater tendency for girls to internalise emotional distress, a pattern also associated with a greater vulnerability to social network addiction as a coping mechanism among girls (Del Brío Ibáñez et al., 2019; Gámez-Guadix et al., 2020).

Notably, this study diverges from findings identifying gender as a moderating variable in the relationship between predictors and NSSI (Obando et al., 2018; Ramírez Gamboa & Restrepo Soto, 2022), in that gender emerges as an independent factor. This suggests that the higher prevalence of NSSI among girls is intrinsically linked to their emotional and behavioural patterns, rather than being solely mediated or moderated by other predictors. This aligns with studies that propose emotional dysregulation and maladaptive coping mechanisms as central to understanding gender differences in NSSI (Bautista Hernández et al., 2022).

Moreover, the inability of maladaptive self-regulatory strategies to predict NSSI in boys, in contrast to their significant role in girls, calls into question gender generalisations. This finding suggests that boys may rely more on externalising behaviours, impulsivity, or substance use to regulate distress, a view supported by studies such as Nock et al. (2006) and Del Brío Ibáñez et al. (2019). Similarly, the stronger association of social network addiction with NSSI in girls points to gender differences in the way digital environments influence emotional regulation and self-injurious behaviours (Carretero et al., 2024; Troya-Fernández et al., 2023). These results highlight the importance of integrating gender-sensitive approaches into both assessments and interventions. For girls, it is crucial to address maladaptive cognitive patterns and emotional vulnerability. For boys, research should focus on alternative mechanisms, such as impulsivity and externalising behaviours, in order to design effective prevention strategies.

Differences between boys and girls suggest important practical implications for clinicians and educators, particularly when designing gender-sensitive interventions to effectively address non-suicidal self-injury (NSSI). Girls demonstrate greater emotional vulnerability, greater reliance on maladaptive self-regulatory strategies such as rumination and self-blame, and greater susceptibility to social network addiction. These factors are strongly associated with the prevalence and severity of NSSI among adolescent girls, as highlighted by studies such as Gámez-Guadix et al. (2020) and Sánchez-Sánchez (2018). In contrast, boys tend to exhibit externalising behaviours and impulsivity as dominant mechanisms, and thus require alternative approaches to mitigate their risk factors, as pointed out by Carretero et al. (2024) and Nock et al. (2006). The gendered nature of these predictors necessitates the development of different strategies tailored to the specific vulnerabilities and coping patterns of each group.

For girls, interventions should focus on addressing maladaptive self-regulatory strategies that play a key role in the maintenance of NSSI. Additionally, the significant role of social network addiction in NSSI among girls highlights the importance of promoting digital literacy and healthy online behaviours. Psychoeducational programmes aimed at managing the emotional impact of social networks and reducing their excessive use can mitigate their role as a maladaptive coping mechanism, as evidenced by Arab and Díaz (2015) and Carretero et al. (2024).

For boys, interventions should prioritise addressing impulsivity and externalising behaviours, which are often used as mechanisms to regulate distress. Addressing the role of substance use as a coping mechanism can reduce associated risks and prevent escalation to more severe forms of self-harm (Del Brío Ibáñez et al., 2019; Obando et al., 2018).

Across genders, integrated screening for emotional symptomatology, self-regulation strategies, and substance use is essential for the early identification of adolescents who are at risk of NSSI. Comprehensive assessments that incorporate these shared risk factors can inform individualised prevention plans that are sensitive to the unique needs of boys and girls (Carretero et al., 2024).

Ultimately, these findings underscore the importance of integrating gender-specific strategies to develop comprehensive prevention and intervention programmes. By addressing the emotional, cognitive, and behavioural patterns associated with NSSI in both boys and girls, these approaches offer a promising avenue toward reducing their prevalence and mitigating their impact during adolescence.

This study has several limitations that should be considered to contextualise its findings and guide future research. The cross-sectional design restricts its ability to establish causal relationships between NSSI and the predictive variables. While predictive and classificatory models offer valuable insights, their applicability remains confined within a correlational framework, according to which significant associations are identified without determining causal pathways. Additionally, the predictive value of these models is limited to the variables included. Factors not examined in this study, such as family dynamics, school support, or broader cultural contexts, could influence the significance and impact of the identified predictors. This underscores the need for longitudinal research to clarify causal mechanisms and temporal relationships more precisely.

In addition, the study’s reliance on self-reported data introduces potential biases, particularly social desirability bias, which may have led participants to under-report NSSI behaviours or emotional symptoms due to stigma. To enhance data reliability, future research should integrate multiple data sources, such as clinical assessments, observational methods, or informant reports. Moreover, the absence of longitudinal data limits the study’s ability to assess the trajectory of NSSI behaviours and the long-term efficacy of emotional regulation strategies. Addressing these gaps through prospective studies would provide a more comprehensive understanding of the developmental course of NSSI and the mechanisms underlying its persistence or remission.

These limitations highlight the need for multi-informant approaches to validate and improve the reliability of findings. The emotional symptomatology measured in this study does not correspond to formal clinical diagnosis, which limits the generalisability of the results. While the predictive and classificatory models are statistically significant, their applicability is restricted to the analysed variables. The inclusion of additional predictors in future studies may result in different weights or interaction effects. Furthermore, the moderate accuracy of the models (69.7%) suggests room for improvement, which could be achieved by incorporating more nuanced variables to increase models’ predictive accuracy.

A key priority for future research should be the implementation of longitudinal studies to establish causal relationships between NSSI and associated factors such as emotional symptomatology, self-regulation strategies, and behavioural or chemical addictions. Such designs would provide deeper insights into the chronology of risk and protective factors, helping to identify critical stages where interventions may be most effective. A prospective approach could also explore how NSSI behaviours evolve from their onset in adolescence to early adulthood, considering the dynamic interplay between individual and contextual factors.

Additionally, future research should adopt a multi-source assessment approach, including structured interviews with participants, reports from family members, validated scales administered by mental health professionals, and, where feasible, direct observations in natural settings. This approach would minimise social desirability bias and increase the external validity of the results. Furthermore, integrating qualitative data would offer a richer understanding of the roles that these variables play in self-injury.

The integration of tracking technologies, such as mobile applications and wearable devices, could enable real-time assessment of self-injurious behaviours and emotional states. To enhance the generalisability of findings, studies should include large, diverse samples selected through stratified random sampling, ensuring representation across socio-economic, cultural, and geographical backgrounds. International multicentre research can further help to identify both universal and culturally specific factors related to NSSI.

Ensuring equitable gender representation, with attention to gender and sexual diversity, is crucial given their differential impact on NSSI prevalence. Including participants with formal clinical diagnoses would provide a clearer understanding of the role of co-morbidities, facilitating tailored interventions for both clinical and non-clinical populations.

Future research should examine contextual factors such as family environment, school dynamics, and social media exposure—particularly the effects of self-injurious content. Additionally, exploring the developmental trajectories of NSSI, differentiating transient from chronic behaviours, remains a key priority.

Emerging technologies, including artificial intelligence, virtual reality, and mobile applications, offer promising tools for early detection and personalised intervention. Evaluating prevention strategies through randomised controlled trials—comparing school programmes, family psychoeducation, and evidence-based therapies like Dialectical Behaviour Therapy and Cognitive Behavioural Therapy—will contribute to more effective public policies and clinical practises.

## Figures and Tables

**Table 1:** Correlation analysis (*N* = 354).

	1	2	3	4	5	6	7
1. Addiction to social networks	1						
2. Substance use	0.16 **	1					
3. Self-control	−0.38 ***	−0.18 **	1				
4. Self-blame	0.21 ***	0.11 *	−0.16 **	1			
5. Rumination	0.26 ***	0.14 **	−0.15 **	0.63 ***	1		
6. Symptomatology	0.33 ***	0.16 **	−0.37 ***	0.43 ***	0.35 ***	1	
7. NSSI (Self-injury)	0.30 ***	0.25 ***	−0.30 ***	0.27 ***	0.24 ***	0.54 ***	1
*Mean*	17.67	1.35	42.27	10.15	11.59	11.42	1.26
*SD*	4.74	0.75	7.84	3.53	4.09	7.55	1.83

* *p* < 0.05; ** *p* < 0.01; *** *p* < 0.001.

**Table 2:** Effects by sex (boys = 183; girls = 171) and self-injury (NSSI = 145; No-NSSI = 201).

	Boys	Girls	*F*	*p*	*d*
	Mean (*SD*)	Mean (*SD*)			
Addiction to social networks	16.56 (4.70)	18.82 (4.52)	11.39	0.001	0.49
Substance use	1.34 (0.75)	1.37 (0.77)	0.06	0.805	0.04
Self-control	43.08 (7.44)	41.40 (8.17)	0.51	0.474	0.22
Self-blame	9.80 (3.56)	10.53 (3.48)	2.00	0.158	0.21
Rumination	10.80 (4.18)	12.44 (3.83)	11.78	0.001	0.41
Symptomatology	9.52 (6.73)	13.44 (7.86)	11.89	0.001	0.54
	NSSI	No-NSSI	*F*	*p*	*d*
	Mean (*SD*)	Mean (*SD*)			
Addiction to social networks	19.21 (4.69)	16.55 (4.47)	19.40	<0.001	0.58
Substance use	1.48 (0.85)	1.26 (0.67)	6.57	0.011	0.29
Self-control	39.76 (7.39)	44.07 (7.67)	24.35	<0.001	0.57
Self-blame	10.93 (3.75)	9.59 (3.27)	9.75	0.002	0.38
Rumination	12.43 (4.20)	11.00 (3.91)	5.61	0.018	0.35
Symptomatology	15.28 (7.75)	8.64 (6.04)	64.19	<0.001	0.96

Note. *d* = Cohen’s effect size.

**Table 3:** Summary of multiple regression analyses predicting self-injury score in the total sample, and in boys and girls separately.

	Total Sample (*N* = 354)			Boys (*N* = 183)			Girls (*N* = 171)		
Variable	*B*	*SEB*	*β*	*B*	*SEB*	*β*	*B*	*SEB*	*β*
Sex	0.40	0.17	0.11 *						
Addiction to social networks	0.03	0.02	0.08	0.02	0.02	0.06	0.03	0.03	0.07
Substance use	0.36	0.11	0.15 **	0.32	0.13	0.17 *	0.49	0.17	0.18 **
Self-control	−0.02	0.01	−0.07	−0.02	0.02	−0.11	−0.02	0.02	−0.06
Self-blame + Rumination	0.01	0.01	−04	−0.03	0.02	−0.15	0.07	0.02	0.20 **
Symptomatology	0.10	0.01	0.42 ***	0.09	0.02	0.42 ***	0.11	0.02	0.40 ***
Constant	−1.03	0.75		0.72	0.89		−1.83	1.16	

*Note.* Sex: 1 = boys, 2 = girls; * *p* < 0.05; ** *p* < 0.01; *** *p* < 0.001.

**Table 4:** Logistic regression analyses predicting self-injury (yes–no) by sex, addictions (social networks and substance use), self-regulation (self-control as an adaptive strategy and self-blame and rumination as maladaptive strategies), and psychological symptomatology.

Predictors	*B*	*SE*	Wald	Exp. (*B*)	95% *CI* (Lower, Upper)
*Total Sample*					
Sex	0.64	0.26	6.24 *	1.90	1.15, 3.13
Addiction to social networks	0.05	0.03	3.30	1.06	0.99, 1.12
Substance use	0.18	0.17	1.14	1.19	0.86, 1.65
Self-control	−0.03	0.02	2.47	0.97	0.94, 1.01
Self-blame + Rumination	−0.00	0.02	0.01	0.99	0.96, 1.04
Symptomatology	0.11	0.02	26.80 **	1.12	1.07, 1.16
Constant	−2.54	1.14	4.97	0.08	
*Boys*					
Addiction to social networks	0.05	0.04	1.33	1.05	0.97, 1.13
Substance use	0.15	0.24	0.39	1.16	0.73, 1.84
Self-control	−0.04	0.03	2.06	0.96	0.91, 1.02
Self-blame + Rumination	−0.06	0.03	3.56	0.95	0.90, 1.00
Symptomatology	0.11	0.03	12.57 **	1.12	1.05, 1.19
Constant	−0.16	1.69	0.01	0.85	
*Girls*					
Addiction to social networks	0.04	0.05	0.97	1.05	0.96, 1.14
Substance use	0.35	0.26	1.84	1.42	0.86, 2.35
Self-control	−0.03	0.03	1.03	0.98	0.93, 1.02
Self-blame + Rumination	0.07	0.03	4.41 *	1.07	1.00, 1.14
Symptomatology	0.11	0.03	14.18 **	1.12	1.05, 1.18
Constant	−2.97	1.62	3.37	0.05	

* *p* < 0.05; ** *p* < 0.001.

## Data Availability

The data used in the study are available upon request to the corresponding author.
